# Impact of Cationic and Neutral Gemini Surfactants on Conidia and Hyphal Forms of *Aspergillus brasiliensis*

**DOI:** 10.3390/ijms19030873

**Published:** 2018-03-15

**Authors:** Anna Koziróg, Bogumił Brycki, Katarzyna Pielech-Przybylska

**Affiliations:** 1 Institute of Fermentation Technology and Microbiology, Faculty of Biotechnology and Food Science, Lodz University of Technology, Wólczańska 171/173, 90-924 Lodz, Poland; katarzyna.pielech-przybylska@p.lodz.pl; 2 Laboratory of Microbiocides Chemistry, Faculty of Chemistry, Adam Mickiewicz University, Umultowska 89b, 61-614 Poznan, Poland; brycki@amu.edu.pl

**Keywords:** gemini surfactants, antifungal activity, conidia germination, ergosterol, scanning electron microscopy

## Abstract

This study investigates the biological activity of two cationic gemini surfactants, hexamethylene-1,6-bis-(*N*,*N*-dimethyl-*N*-dodecylammonium bromide) C6 and pentamethylene-1,5-bis-(*N*,*N*-dimethyl-*N*-dodecylammonium bromide) C5, and their two neutral analogs, hexamethylene-1,6-bis-(*N*-methyl-*N*-dodecylamine) (A6) and pentamethylene-1,5-bis-(*N*-methyl-*N*-dodecylamine) (A5). Experiments were performed with *Aspergillus brasiliensis*, which is used in the standard tests for biocides. The minimal inhibitory concentration (MIC) values for conidia and mycelium were determined using the dilution method. The viability of the conidia was evaluated using the plate count method. The dry mass of the mycelium was determined using the thermogravimetric method. Ergosterol was extracted from the mycelium and evaluated by gas chromatography. The effect of gemini surfactants on fungal morphology was observed using scanning electron microscopy. Cationic gemini surfactants were found to be active at lower concentrations compared to their non-ionic analogues, rapidly reducing the total number of conidia that were able to grow. They also decreased both the ergosterol content in the mycelium and its dry weight. These results suggest that cationic gemini surfactants C6 and C5 could have a wide range of practical applications as active compounds. However, it should be remembered that usage at too low concentrations, below the MIC, will only lead to short-term disturbances in the development of conidia and mycelium.

## 1. Introduction

Gemini surfactants are amphiphilies composed of two hydrophilic head groups and two hydrophobic hydrocarbon tails, linked by a spacer at the head groups or nearby. They cover a wide hydrophilic-lipophilic balance (HLB) range, and are characterized by very low critical micelle concentrations (CMC), low surface tension (γ) and low minimal inhibitory concentrations (MIC) [1,2,3,4,5]. The unique properties of gemini surfactants make them very useful as ingredients in detergents, cosmetics and personal care products, as additives in paints and coatings, as biocides, in materials science, for organic synthesis, in pharmaceuticals, in textiles, for enhanced oil recovery, in nanotechnology, in the petroleum industry and many other areas [6]. Until recently, research on the impact of gemini surfactants on microorganisms has focused mainly on bacteria and yeast [7,8,9]. With regard to moulds, experiments have been conducted only to determine minimal inhibitory concentration (MIC) values [10,11]. According to the current methodology [12,13], conidia are used to evaluate antimicrobial activity. However, moulds possess two morphological forms—conidia and mycelium. As Plumridge et al. [14] and van de Sande et al. [15] have shown, these forms show different types of sensitivity to biocides.

In procaryotic or eucaryotic cells, there are several areas susceptible to the activity of biocides, including the cell membrane [16,17], which is composed predominantly of ergosterol. Ergosterol plays a very important role in cellular membranes—for instance, it sustains the proper functioning of the cell by transporting substances. The ergosterol method is used to estimate the level of mould infestation in buildings [18,19,20,21] and to evaluate the risk of food contamination by toxin-forming moulds [22,23]. This method is also recommended for the monitoring of yeast and moulds in soil during composting or bioremediation [24,25]. Ergosterol can be detected using high performance liquid chromatography [26], gas chromatography with a flame ionization detector [27] or mass spectrometry [28], or by spectrophotometric UV [19].

The aim of the present study is to describe the antifungal activity against *Aspergillus brasiliensis* ATCC 16404 of cationic gemini surfactants hexamethylene-1,6-bis-(*N*,*N*-dimethyl-*N*-dodecylammonium bromide) (C6) and pentamethylene-1,5-bis-(*N*,*N*-dimethyl-*N*-dodecylammonium bromide) (C5), as well as that of their neutral analogues, hexamethylene-1,6-bis-(*N*-methyl-*N*-dodecylamine) (A6) and pentamethylene-1,5-bis-(*N*-methyl-*N*-dodecylamine) (A5). Particular attention is given to conidia and hyphae morphology, growth and ergosterol content.

## 2. Results

### 2.1. Minimal Inhibitory Concentration

The effectiveness of cationic (C5, C6) and neutral (A5, A6) gemini surfactants on *Aspergillus brasiliensis* ATCC 16404 treatment was examined by determining the minimal inhibitory concentration (MIC) for conidial growth. Table 1 presents the results of MIC values for 4 tested compounds and different morphological forms.

The minimum concentrations at which the cationic surfactants inhibited conidia formation were three-fold lower (0.12 mM) compared to their neutral analogues (0.38 mM). A substantial difference can be observed in the case of mycelium. The ionic compounds suppressed the formation of mycelium at concentrations nearly a 100-fold lower (0.31 mM) than the MICs of the neutral compounds (25–30 mM). The MIC values of the four tested compounds clearly show that conidia are more sensitive than the other morphological forms of mould. The MICs of alkanediyl-α,ω-bis(dodecyldimethyloammonium bromides) (C5, C6) for conidia were 2.5-fold lower than those for mycelium, while the MICs of the neutral surfactants were over 60-fold lower for conidia than for mycelium (Table 1).

### 2.2. Viability of A. brasiliensis Conidia in the Presence of Microbiocides

In the first stage of the study, the viability of planktonic cells was analysed in medium containing surfactants.

Table 2 displays the number of viable conidia (conidia/mL) after treatment with gemini surfactants. The application of gemini surfactants at ½ MIC concentrations after 4 h of treatment caused a statistically significant (*p* < 0.05) reduction in the number of conidia of 0.8 log. However, after 8 h of biocide activity, their number was reduced significantly, by up to a maximum of 1.3 log (Table 2). The formation of mycelium was detected after 24 h of treatment in the control sample and the sample with ½ MIC. When the concentration of cationic and neutral surfactants was raised to the MIC value, a reduction in the number of conidia was observed of between 1.1 to 2.3 log after 4 h, and of 1.5 to 3.3 log after 8 h, when compared to the non-treated sample. After 24 h of treatment with cationic compounds, the formation of conidia was entirely suppressed. By comparison, after 24 h of treatment with neutral surfactants only several conidia still showed the ability to germinate. Hexamethylene-1,6-bis-(*N*,N-dimethyl-*N*-dodecylammonium bromide) C6 was found to be the most reactive. Its application at 2 MIC concentration prevented the germination of conidia soon after 8 h. In contrast, the other cationic surfactant, C5, caused a 4.3 log reduction in the number of viable conidia. The least effective compound was pentamethylene-1,5-bis-(*N*-methyl-*N*-dodecylamine)– A5. Active germination of conidia was detected even after 24 h. Long-term exposure to all gemini surfactants resulted in *A. brasiliensis* losing the ability to germinate after 48 h. Similar results were obtained by SEM analysis.

### 2.3. Dry Weight and Ergosterol in Mycelium after GS Treatment

**Figure 1:**
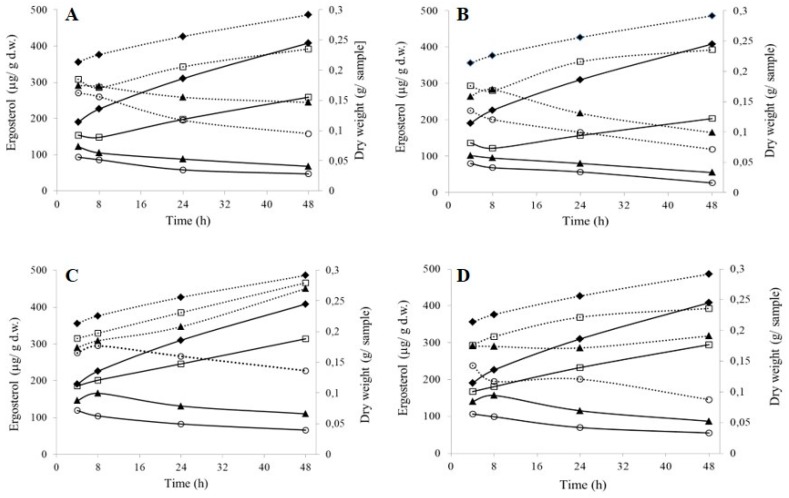
Dry weight (black, solid line) and ergosterol (dotted line) in mycelium following treatment with: cationic gemini surfactants (**A**) pentamethylene-1,5-bis-(*N*,*N*-dimethyl-*N*-dodecylammonium bromide) C5 and (**B**) hexamethylene-1,6-bis-(*N*,*N*-dimethyl-*N*-dodecylammonium bromide) C6; or with non-ionic gemini surfactants (**C**) pentamethylene-1,5-bis-(*N*-methyl-*N*-dodecylamine) A5 and (**D**) hexamethylene-1,6-bis-(*N*-methyl-*N*-dodecylamine) A6. The results are displayed for ½ MIC (square marker), MIC (triangle marker), 2 MIC (circle marker) and the control sample without the addition of surfactant (rhombus marker).

Figure 1 shows changes in dry weight and ergosterol for mycelium over time, following treatment with the two cationic gemini surfactants or their neutral analogues.

While observing changes in the dry weight of the mycelium, it was noted that after treatment with the surfactants the growth of the strains was inhibited, even at the lowest concentrations (½ MIC). The neutral surfactants A5 and A6 were least effective, as after 4 h of treatment there were no clear differences in mycelial dry mass compared to the samples without biocide. As the concentrations of all the compounds was increased (MIC and 2 MIC), the mycelial dry weight gradually decreased, which may indicate that they had lethal impact (Figure 1). The greatest difference between the treated samples and the control sample was detected for cationic surfactant C6, regardless of the concentration (Figure 1B). The dry weight was half that of the control sample after 48 h at ½ MIC = 0.155 mM, while at 2 MIC the dry weight was 15 times lower than that of the control sample.

Another important marker associated with the formation of mycelium is ergosterol. Exposure to gemini surfactants, both cationic and neutral, at ½ MIC had little impact on ergosterol content, which was comparable to that in the control sample (Figure 1, dotted line). When the concentration was two times higher, the content of ergosterol was lower only in the culture containing cationic surfactants (Figure 1A,B). The use of neutral surfactants at MIC concentration induced an increase in ergosterol content over time (Figure 1C,D). In the case of compound A5, this process was nearly the same as that observed in the control sample. It is noteworthy that the synthesis of ergosterol was hindered when the concentration of all the studied compounds reached 2 MIC.

A comparison of the content of ergosterol and the mycelial dry mass over 48 h shows a strong linear correlation (>0.8). However, a negative Pearson’s coefficient was determined for the cultures containing neutral gemini surfactants at MIC (Table 3). Therefore, it can be said that the lower the mycelial dry mass, the higher ergosterol content.

The effect of A5 and A6 compounds on mycelium growth was also examined. The results revealed that these compounds did not increase the biomass yield, while an increase was observed in the concentration of ergosterol. Moreover, the mycelium had stiffer membranes, which indicates that they were defending against the penetration of the surfactants into the cells.

### 2.4. Ergosterol Binding Assay

Binding test results, showing the ability of gemini surfactant to bind to ergosterol, are presented in Table 4. When the activity of the gemini surfactants was caused by binding to ergosterol, the exogenous ergosterol was prevented from binding to the fungal membrane’s ergosterol. This caused an increase in the MIC value for the sample with exogenous ergosterol in comparison to the control.

In the binding assay, the MICs were two times lower in the presence of ergosterolin in the case of neutral gemini surfactants and three time higher in the case of their cationic analogues, in comparison to the control. This may indicate that cationic gemini surfactants, like several monomeric quaternary ammonium salts [29], possess better affinity for ergosterol.

### 2.5. Effects of GS on Fungal Morphology by Scanning Electron Microscopy

**Figure 2:**
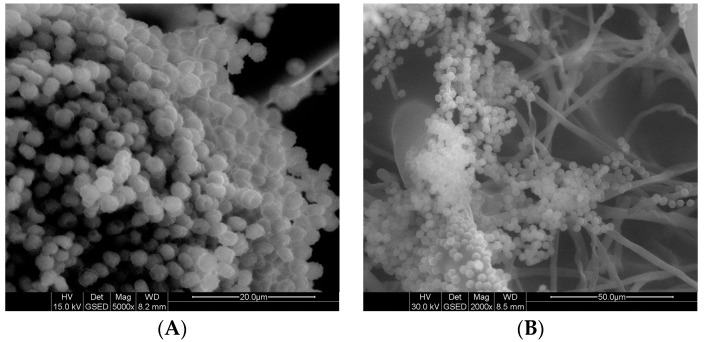

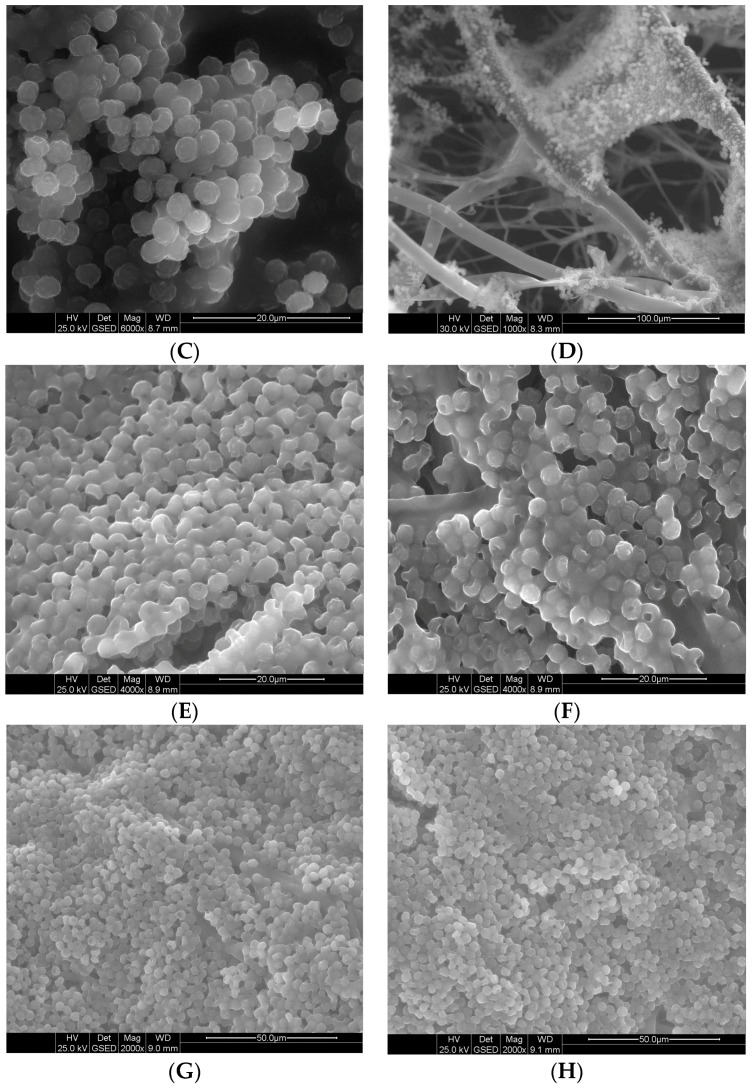
Figure **2.** Changes in morphology of conidia: control sample without biocide after 4 h (**A**) and 24 h (**B**); with cationic GS C6 at ½ MIC after 4 h (**C**) and 24 h (**D**); sample with cationic GS C6 at MIC after 24 h (**E**); sample with non-ionic GS A6 at MIC after 24 h (**F**); sample with cationic GS C6 at 2 MIC after 24 h (**G**); sample with non-ionic GS A6 at 2 MIC after 24 h (**H**).

Analysis of SEM images revealed an absence of structural changes both in non-treated conidia of *A. niger* and with surfactants at ½ MIC. After 4 h, swelling of the conidia was observed (Figure 2A,C). After 24 h, however, a fully structured mycelium was detected (Figure 2D), similar to that in the control sample without biocide (Figure 2B). After 24 h of exposure to cationic C6 (Figure 2E,G) and neutral A6 (Figure 2F,H) gemini surfactants at MIC and 2 MIC, the conidia were conglomerated, which made the isolation of individual structures impossible.

**Figure 3:**
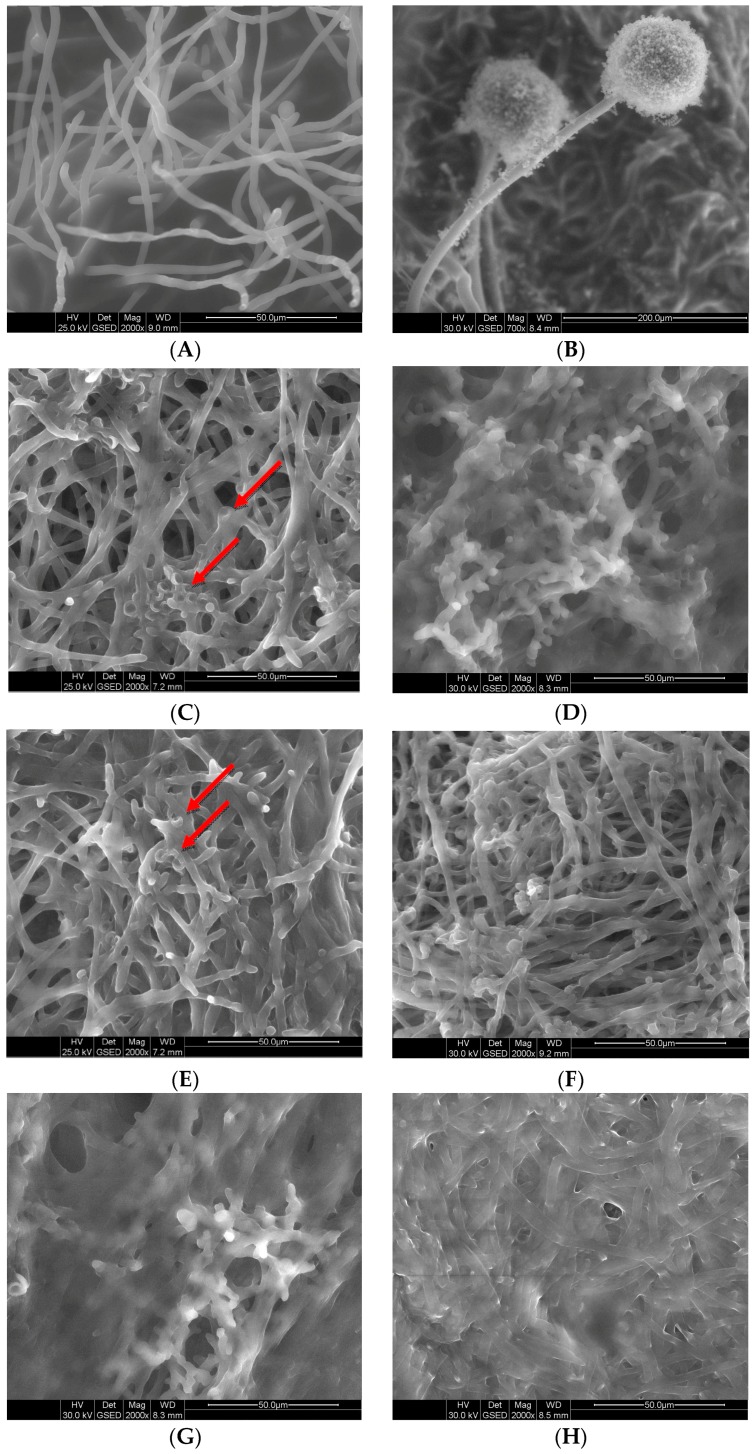

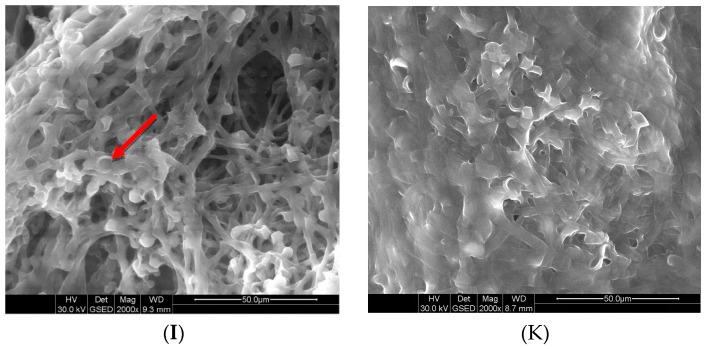
Two days old mycelium: sample with cationic GS C6 at ½ MIC after 24 h (**A**); sample without GS after 24 h (**B**); sample with cationic GS C6 at MIC after 4 h (**C**) and 24 h (**D**); sample with non-ionic GS A6 at MIC after 4 h (**E**) and 24 h (**F**); sample with cationic GS C6 at 2 MIC after 4 h (**G**) and 24 h (**H**); sample with non-ionic GS A6 at 2 MIC after 4 h (**I**) and 24 h (**K**).

Figure 3 displays morphological changes in mycelium induced by exposure to the gemini surfactants. In the non-treated mycelium and the mycelium treated with surfactants at ½ MIC, well-developed hyphae can be observed (Figure 3A). In the control sample, conidiophores can also be seen (Figure 3B). Disk-like depressions are visible on the surface of hyphae of the mycelium after 4 h of activity by the cationic (C6) and neutral (A6) compounds at MIC and 2 MIC (Figure 3C,E,I, red arrow). The most severe damage was registered in the mycelium after 4 h of cationic C6 gemini surfactant activity at 2 MIC (Figure 3G). After 24 h, further degradation of the hyphae appeared (Figure 3D,F,H,K). A conglomerated structure formed, which was especially pronounced after the application cationic gemini surfactants (Figure 3D,H). With compound A6 at MIC, single hyphae were detected, but they also consolidated into larger fragments.

## 3. Discussion

Fungi are an often overlooked object of research, especially in studies of the effects of biocide treatment. Until recently, investigations into ‘antifungal activity’ focused on yeast, especially *Candida* sp. [12,30]. However, numerous genera now show decreasing sensitivity to biocides, due to their inappropriate use. Damage caused by fungi is a growing problem, with increasing economic consequences [31]. Therefore, newly developed compounds are needed, with inhibitory properties even at low concentrations. Gemini surfactants are considered to be the best candidates for this purpose.

The chemical structure of gemini surfactants has a major influence on their MIC values. In our study, two cationic compounds were used, containing the C12 alkyl chain and 5–6 methylene groups in the spacer. According to Shukla and Tyagi [3] and Brycki et al. [5], this chemical structure helps to provide the lowest MIC values. In comparison to neutral surfactants with identical chain lengths, the cationic gemini surfactants were active at much lower concentrations. The reason for this may be that policationic compounds can easily interact with charged cell surfaces. Moreover, they can increase membrane fluidity and consequently lead to cell death [32,33,34,35]. The results presented in our study are similar to those reported for yeast by Obłąk et al. [36]. The MIC values for both cationic and neutral surfactants were comparable to those for gemini quaternery ammonium salts with a betaine-based ester type alkyl chain containing the same number of methylene groups. These results clearly demonstrate that the MIC values for fungi are from several to several tens of times higher than those for bacteria, as reported previously by Koziróg and Brycki [37], as well as by Shirai et al. [38].

Comparison of the two morphological forms revealed that mycelium was less sensitive to biocides than conidia. This confirms findings by van de Sande et al. [15], Lass-Flörl et al. [39] and Guarro et al. [40] for different compounds. Mycelium develops vertically [41], hence apical parts of the fungal hyphae are more susceptible to biocides, while ‘older’ fragments, which are often metabolically inactive, remain intact. It is therefore useful to examine the activity of antifungal compounds on both morphological forms. On one hand, mycelium that grows on the surface can be macroscopically examined and mechanically removed prior to disinfection. On the other hand, several hyphae, more resistant to biocide than conidia, may remain on the surface. A serious problem occurs when mycelium forms a biofilm, especially inside narrow pipes or on historic paint coatings, where it is hard to remove. Another example within living organisms is pulmonary aspergillosis. As van de Sande et al. note, when histopathological examination of tissue detects fungal hyphae, an antimicrobial agent should be taken at high concentrations [15]. Too low concentrations, also known as sub-MICs, may lead to the adaptation of microorganisms and development of resistant strains [42,43]. This was confirmed in the present study, when cationic and neutral gemini surfactants at ½ MIC caused temporary inhibition of conidia and mycelium.

The activity of biocides induces several defence mechanisms in cells, which may have a phenotypic character or be related to genes. One example is the sealing of the cell wall or of a membrane in response to antifungal agents, reducing the uptake of fungicide. The vast majority of antifungal agents act mainly on fungal membranes, which are exposed to the activity of chemicals in the environment. One group, including polyenes and quaternary ammonium salts, binds to the lipid bilayer, causing disruption of the fungal cell membrane and leakage of the cytoplasmic contents. Another group, including azoles, allylamines and morpholines, reacts with ergosterol itself or disrupts ergosterol biosynthesis, which has an impact on membrane synthesis [44,45,46,47].

Ergosterol is a basic sterol for the formation of cellular membranes, but it may also be present in the internal structures of cells, such as in mitochondrial membranes. It can serve as a marker for the presence of moulds [21,26,48]. It does not always correlate with the growth of mycelium and dry weight. The relationship between dry mass and ergosterol showed a clear linear Pearson correlation coefficient. The concentration of this sterol may increase significantly as a consequence of biocidal activity. An elevated content of ergosterol protects cells against adverse environmental conditions and induces stiffening of membranes [49]. We observed this mechanism after applying non-ionic gemini surfactants. Nonetheless, if a biocide does not affect the synthesis of ergosterol or is not connected with that process, the concentration of ergosterol will not be lowered immediately even if the mycelium dies. Mille-Lindblom et al. [50] and Gutarowska et al. [27] report slow degradation of ergosterol and its presence in appreciable concentrations in the absence of living fungi. Membranous structures containing ergosterol may still be present even after cell death, as confirmed in our previous research [51].

The present study proves that cationic gemini surfactants are more active at lower concentrations than their non-ionic analogues, leading to extensive destruction of conidia and mycelium. Moreover, these compounds rapidly reduce the total number of conidia that are able to grow, decreasing ergosterol content in mycelium and resulting in a loss of dry weight. These properties make them suitable as antifungal agents for disinfection. 

## 4. Materials and Methods

### 4.1. Strain and Growth Condition

*Aspergillus brasiliensis* ATCC 16404 (previously *A. niger*), the species of fungus used in this study, is recommended as a candidate quality control microorganism for an antifungal susceptibility test (EN 1650). The strain was stored on Malt Extract Agar (MEA) (MERCK, Darmstad, Germany) slants at 4 °C. Prior to each experiment, the strain was subcultured in MEA medium at 28 °C for 4–5 days until the conidia were fully mature. Spore suspensions were prepared by washing the conidia from the agar slants using deionized sterilized water with 0.1% Tween 80, and then stirred. The spore concentrations in the initial water suspensions were checked using a Thoma chamber and the concentrations were adjusted to 1.0–2.0 × 10^7^ conidia/mL.

### 4.2. Gemini Surfactants

The antimicrobial agents used in the study were the gemini surfactants (GS) hexamethylene-1,6-bis-(*N*,N-dimethyl-*N*-dodecylammonium bromide) (C6) and pentamethylene-1,5-bis-(*N*,N-dimethyl-*N*-dodecylammonium bromide) (C5), as well as their neutral analogues, heksyleno-1,6-bis(dodecylometyloamina) (A6) and pentyleno-1,5-bis(dodecylometyloamina) (A5). The gemini surfactants were obtained according to procedures described in the literature [5,52].

### 4.3. Minimal Inhibitory Concentration (MIC)

The MIC values were determined by the dilution method. The fungal strain was cultured in 16 mL of Malt Extract Broth (MEB) medium (MERCK, Darmstad, Germany), inoculated with 2 mL of conidia and incubated for 48 h at 28 °C. Aliquots of 2 mL of the cationic gemini surfactants (0.05–15 μM/mL) and neutral gemini surfactants (0.75–150 μM/mL) were added. The samples were incubated for another 48 h. A sample without biocide was treated as a control. After 24 and 48 h, the morphology of the mycelium was evaluated macroscopically. The MIC values were defined as the lowest concentrations of the compounds at which the development of mycelium was inhibited in comparison to the control sample [5]. The mycelium was fully submerged in medium. The MIC values for conidia were determined according to the method described by Koziróg and Brycki [37]. The analysis was performed in triplicate.

### 4.4. Viability of A. brasiliensis Conidia in the Presence of Microbiocides

The growth of conidia was evaluated in a liquid medium of 17.8 mL MEB with the addition of 2 mL of gemini surfactant at ¼ MIC, ½ MIC and MIC concentrations, previously designated. In the control sample, 2 mL of sterile, distilled water was used instead of antimicrobial agents. Each test flask was inoculated with 0.2 mL of standardized conidia suspension to obtain a conidia level of 1–2 × 10^6^ cfu/mL. The samples were incubated at 28 °C for 2 days. After 4, 8, 24 and 48 h, 1 mL of each mixture was transferred to 9 mL of 0.85% (*w*/*v*) saline with a mixture of neutralizers (5% Tween 80, 2% lecithin and 0.5% sodium thiosulfate) [53]. Viabile cells were determined using the conventional plate count method and MEA agar medium (MERCK, Darmstad, Germany). After incubation at 28 °C for 48 h, the colonies of *A. brasiliensis* were counted. The analysis was performed in triplicate.

### 4.5. Dry Weight of Mycelium after GS Treatment

The samples were prepared analogously to those used for MIC determination. Each sample was then supplemented with gemini surfactants at ½ MIC, MIC or 2 MIC. A sample without gemini surfactants served as the control. In order to measure the fungal biomass after 48 h of incubation at 28 °C, the mycelium was filtered through filter paper and dried to a constant weight. The mycelium was placed in a moisture analyzer and the dry weight determined using the thermogravimetric method. The results were expressed as dry weight (in grams) per medium volume. The experiment was performed in triplicate.

### 4.6. Content of Ergosterol in Mycelium after GS Treatment

Extraction of ergosterol was performed on untreated mycelium and mycelium exposed to gemini surfactants at ½ MIC, MIC and 2 MIC concentrations, according to the methodology developed by Seitz et al. [54] and further modified by Gutarowska and Żakowska [19]. The ergosterol content was determined by gas chromatography (GC). Chromatographic analysis was carried out using a GC apparatus (Agilent 7890A, Agilent Technologies, Santa Clara, CA, USA) with a mass spectrometer (Agilent MSD 5975C, Agilent Technologies, Santa Clara, CA, USA). A HP-5 MS capillary column (30 m × 0.25 mm × 0.25 µm) was used to separate the compounds. The GC oven temperature was programmed to increase from 150 °C (1 min) to 305 °C (6 min) at a rate of 30 °C/min. Helium was used as the carrier gas at a flow rate of 1.2 mL/min. The temperature of the injector was kept at 290 °C. Injections (1 µL) were made in the split mode (1:5). The MS conditions were as follows: ion source temperature 230 °C; transfer line temperature 300 °C; quadrupole temperature 150 °C; ionization energy 70 eV. The MSD was operated in the selected-ion monitoring mode (SIM) with the *m*/*z* 337 and 363 ions used for ergosterol quantification. As an internal standard, 7-hydrocholesterol was used to monitor the instrument response and retention time stability.

Quantitative analysis was performed using Agilent MassHunter software (version B 07.00, Agilent Technologies, Santa Clara, CA, USA). The detection limit of ergosterol was LOD = 0.0603 µg/mL. The ergosterol concentrations were expressed as micrograms of ergosterol per gram of dry weight. The analysis was performed in triplicate. 

### 4.7. Ergosterol Binding Assay

To find out whether the gemini surfactants bind to sterols in fungal membranes, their MIC values substances against conidia of *A. brasiliensis* were determined in the presence or absence of 400 μg/mL ergosterol (Sigma-Aldrich, St. Louis, MO, USA). The MIC values for conidia were determined according to the method described above. The binding assay showed the ability of the surfactants to bind to ergosterol [55].

### 4.8. Effects of GS on Fungal Morphology by Scanning Electron Microscopy

The morphological changes in the conidia and mycelium were observed 4 and 24 h after the addition of the gemini surfactants to the cultures. Samples were analyzed using a Quanta 200 (FEI Co., Hillsboro, OR, USA) scanning electron microscope. Tests were performed in environmental mode, which allows samples to be studied in their natural state.

### 4.9. Mathematical Calculation

The mean results from three independent experiments were calculated, together with their standard deviations. Statistical differences in the data were compared using a one-way repeated measures analysis of variance (ANOVA; OriginPro 9.2.214, OriginLab Corp., Northampton, MA, USA). Statistical significance was set at 5% (*p* < 0.05).

The linear correlation coefficient between two variables, dry mass and ergosterol, was measured. The Pearson correlation coefficient was determined using Excel (Microsoft Office 2013). The Pearson correlation has a value between +1 and −1, where −1 is a total negative linear correlation, 1 is total positive linear correlation and 0 is no linear correlation.

## 5. Conclusions

In this study, the cationic gemini surfactants hexamethylene-1,6-bis-(*N*,*N*-dimethyl-*N*-dodecylammonium bromide) C6 and pentamethylene-1,5-bis-(*N*,*N*-dimethyl-*N*-dodecylammonium bromide) C5 were found to exhibit high antimicrobial efficacy against *Aspergillus brasiliensis*—one of the moulds least sensitive to the action of antimicrobial agents. Even at a very low concentration of 0.12 mM, these compounds reduced the number of viable conidia. The mycelium was less sensitive to biocides than the conidia. The cationic GS inhibited development of mycelium at 0.31 mM. A reduction in dry weight and a decrease in ergosterol were observed. However, when investigating the effects of antimicrobial agents, it should be noted that ergosterol is not necessarily a good indicator of the effectiveness of antifungals on moulds.

The results of this study suggest that cationic gemini surfactants are superior to their non-ionic analogues and could have a wide range of practical applications as active compounds.

## Figures and Tables

**Table 1:** Minimal inhibitory concentration (mM) of cationic (C) and neutral (A) gemini surfactants for *A. brasiliensis* ATCC 16404 conidia and mycelium.

Biocide	Conidia	Mycelium
C5	0.12	0.31
C6	0.12	0.31
A5	0.38	30
A6	0.38	25

**Table 2:** Viability of conidia *A. brasiliensis* ATCC 16404 (log conidia/mL) in medium with microbiocides.

Biocide Concentration (mM)		Biocide	Time (h)			
			4	8	24	48
control	0.00	K	6.20 ± 0.05	6.18 ± 0.10	mycelium	Mycelium
½ MIC	0.06	C5	5.93 ± 0.53	5.46 ± 0.15 ^#^	mycelium	Mycelium
	0.06	C6	5.51 ± 0.37 ^#^	4.18 ± 0.11 ^#^	mycelium	Mycelium
	0.19	A5	5.90 ± 0.60	5.87 ± 0.22 ^#^	mycelium	Mycelium
	0.19	A6	5.92 ± 0.10	5.71 ± 0.20 ^#^	mycelium	Mycelium
MIC	0.12	C5	4.49 ± 0.03 ^#^	4.72 ± 0.13 ^#^	nd	nd
	0.12	C6	3.87 ± 0.02 ^#^	2.91 ± 0.23 ^#^	nd	nd
	0.38	A5	5.11 ± 0.03 ^#^	2.73 ± 0.16 ^#^	1.64 ± 0.05 ^#^	nd
	0.38	A6	4.92 ± 0.03 ^#^	4.20 ± 0.31 ^#^	1.04 ± 0.02 ^#^	nd
2 MIC	0.24	C5	2.91 ± 0.10 ^#^	1.85 ± 0.08 ^#^	nd	nd
	0.24	C6	2.38 ± 0.24 ^#^	nd	nd	nd
	0.76	A5	4.30 ± 0.32 ^#^	2.97 ± 0.15 ^#^	1.25 ± 0.01 ^#^	nd
	0.76	A6	3.08 ± 0.28 ^#^	2.48 ± 0.21 ^#^	nd	nd

MIC—minimal inhibitory concentration; nd—not detected in 1 mL; K—control sample; ^#^ Reduced value of log 10 differed significantly from the control without surfactant (*p* < 0.05).

**Table 3:** Pearson’s correlation coefficient (*r*) for ergosterol concentration and mycelial dry weight content.

Biocide Concentration (mM)		Biocide	Correlation Coefficient *r*
K	0.00	-	0.999
½ MIC	0.06	C5	0.987
	0.06	C6	0.947
	0.19	A5	0.999
	0.19	A6	0.966
MIC	0.12	C5	0.958
	0.12	C6	0.958
	0.38	A5	−0.869
	0.38	A6	−0.750
2 MIC	0.24	C5	0.996
	0.24	C6	0.990
	0.76	A5	0.808
	0.76	A6	0.837

**Table 4:** Effect of exogenous ergosterol on MICs of cationic and neutral gemini surfactants for *A. brasiliensis* conidia.

Biocide	Control	With Ergosterol
C5	0.12	0.36
C6	0.12	0.36
A5	0.38	0.19
A6	0.38	0.19

## Abbreviations

GS	Gemini surfactant
MIC	Minimal Inhibitory Concentration
HLB	Hydrophilic-Lipophilic Balance
ATCC	American Type Culture Collection
